# Dynamic Calibration and Verification Device of Measurement System for Dynamic Characteristic Coefficients of Sliding Bearing

**DOI:** 10.3390/s16081202

**Published:** 2016-07-30

**Authors:** Runlin Chen, Yangyang Wei, Zhaoyang Shi, Xiaoyang Yuan

**Affiliations:** Key Laboratory of Education Ministry for Modern Design and Rotor-Bearing System, Xi’an Jiaotong University, Xi’an 41049, China; wyytwm1314@stu.xjtu.edu.cn (Y.W.); zhaoyang-szy@stu.xjtu.edu.cn (Z.S.); zonghezu_xjtu@163.com (X.Y.)

**Keywords:** sliding bearing, dynamic characteristics, stiffness and damping coefficients, measurement system, calibration

## Abstract

The identification accuracy of dynamic characteristics coefficients is difficult to guarantee because of the errors of the measurement system itself. A novel dynamic calibration method of measurement system for dynamic characteristics coefficients is proposed in this paper to eliminate the errors of the measurement system itself. Compared with the calibration method of suspension quality, this novel calibration method is different because the verification device is a spring-mass system, which can simulate the dynamic characteristics of sliding bearing. The verification device is built, and the calibration experiment is implemented in a wide frequency range, in which the bearing stiffness is simulated by the disc springs. The experimental results show that the amplitude errors of this measurement system are small in the frequency range of 10 Hz–100 Hz, and the phase errors increase along with the increasing of frequency. It is preliminarily verified by the simulated experiment of dynamic characteristics coefficients identification in the frequency range of 10 Hz–30 Hz that the calibration data in this frequency range can support the dynamic characteristics test of sliding bearing in this frequency range well. The bearing experiments in greater frequency ranges need higher manufacturing and installation precision of calibration device. Besides, the processes of calibration experiments should be improved.

## 1. Introduction

Research on the dynamic characteristics of sliding bearing became serious in the 1980s [1,2], and it is generally understood that the dynamic characteristics of sliding bearing are important to the stability of the rotor [3]. Under the linear theory, the dynamic relationships between the motivation and response of bearings are usually described by stiffness and damping coefficients [4,5,6,7]. The dynamic characteristics measurement of sliding bearing aims to obtain the inner relationships between the motivation and response by analyzing the dynamical behavior data, which is the stiffness and damping coefficients of sliding bearings [8,9,10]. There are many methods to measure the dynamic characteristics, including time domain methods and frequency domain methods, such as dynamic excitation method, influence coefficient method, hammering method, harmonic scanning method, and so on [11,12,13,14,15]. The measurement systems are different, but they all include force sensors, displacement sensors, data collectors and other auxiliary components. Scholars at home and abroad have done a lot of theoretical and experimental research on the dynamic characteristics measurement of sliding bearing [16,17,18,19,20]. However, the test results are usually very different from the theoretical results [20,21,22,23], and the repeated accuracy of the test is not high.

The measurement system incurs test errors itself. Some theoretical research shows that the 1° phase error of displacement test will cause over 10% identification errors of stiffness and damping coefficients [24]. So the calibration of the measurement system is very important. There are two types of calibration methods: static calibration and dynamic calibration. In the static calibration, the functional relationships between the input signals and the output signals are given by experiments [25]. The static calibration methods of different sensors are almost the same in principle [26,27,28], and the calibrations of sensors are usually accomplished by the manufacturing factories. The high quality testing elements have a smaller test error after calibration. Generally, only amplitude data are given because the phase data are not detected or the test data are not accurate. This type of calibration only aims at the sensor itself, and cannot eliminate the test errors of other elements and error caused by connection of components [29,30]. In the dynamic calibration, multiple test channels are jointly calibrated at the same time, and the test errors of the whole measurement system can be eliminated to some extent [31,32,33]. The measurement system is calibrated by using the vibration of a single quality system in the calibration method of suspension quality [34]. However, the movements of mass point cannot simulate the bearing vibration, which causes the ranges of amplitude and frequency after high accuracy calibration to be small. The measurement system will cause large errors when used as the bearing test in a wide range of amplitudes and frequencies.

A new device for implementing the dynamic calibration method is proposed in this paper. It uses the movements of spring-mass system, and the movements can simulate the vibration of the bearing to some extent. Then the amplitude and frequency ranges of measurement system in calibration condition are basically the same with that in a working condition. A measurement system for dynamic characteristic coefficients of the sliding bearing has been calibrated in this new calibration device, and the calibration data are verified to be effective by simulation experiment.

## 2. Identification Theories and Methods for Dynamic Characteristic Coefficients of Sliding Bearing

**Figure 1:**
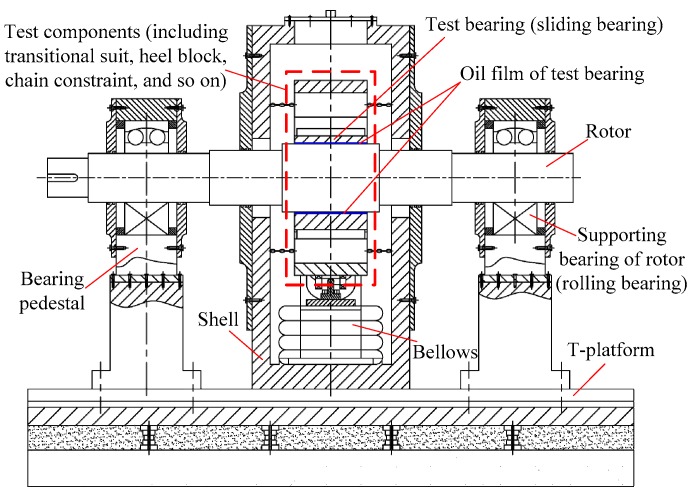
Structure diagram for inversion test bed of sliding bearing (getting rid of the excitation device for dynamic vibration).

Taking the inversion test bed of sliding bearing as an example [35], the structure diagram is shown in Figure 1. The rotor is supported by two rolling bearings, and it can rotate around the horizontal axis. The test bearing (which is a sliding bearing) is installed on the rotor. It only has the freedoms of translational motions in horizontal and vertical directions, and other freedoms of motions are constrained by chains. There is an oil film between the rotor and test bearing. The test components are installed outside the bearing pedestal, through which the dynamic excitation forces act on the test bearing. Then the bearing vibrates, and so does the rotor because of the forces transferred through the oil film.

**Figure 2:**
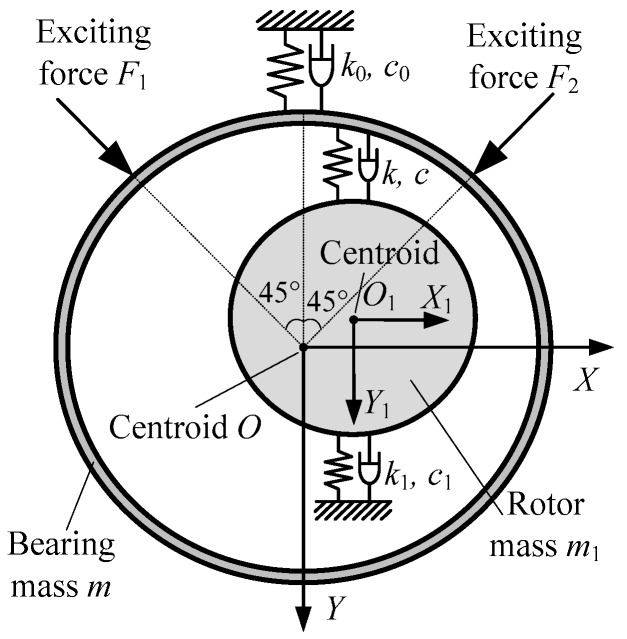
Dynamical model of the test bearing system.

The dynamical model of the inversion test bed system is shown in Figure 2. *F*_1_, *F*_2_ are the exciting forces in two directions respectively. *X*, *Y* are respectively the absolute displacements of bearing in two coordinate directions with respect to static balance position. *X*_1_, *Y*_1_ are respectively the absolute displacements of the rotor in two coordinate directions with respect to static balance position. *X*_2_, *Y*_2_ are respectively the relative displacements of the bearing in two coordinate directions with respect to the rotor.

If the vectors of exciting forces pass the geometric center of the bearing, and the bearing moves in a plane, then the differential equations of motion for the system are as follows:

(1) {m[X¨Y¨]+k0[XY]+c0[X˙Y˙]+k[X2Y2]+c[X˙2Y˙2]=[2222]F1+[−2222]F2m1[X¨1Y¨1]+k1[X1Y1]+c1[X˙1Y˙1]+k[−X2−Y2]+c[−X˙2−Y˙2]=0

where *k*_0_, *c*_0_, *k*_1_, *c*_1_ are all the 2 × 2 matrixes, and *k*_0_, *c*_0_ are the coupling stiffness and damping coefficients. *k*_1_, *c*_1_ are the supporting stiffness and damping coefficients of rotor, and can be obtained by the stiffness and damping superposition of the two supporting bearings. *k*, *c* are the stiffness and damping coefficients of bearing oil film. *m* is the mass of test bearing. *m*_1_ is the mass of rotor.

In Equation (1), the masses are known, and the exciting forces and displacements can be acquired by sensors. So the eight stiffness and damping coefficients of the bearing can be solved theoretically by only four groups of forces and displacements test data. However, in practice, the experiment system is disturbed by environmental active disturbance forces, temperature drift of measurement system, roundness of journal, and so on, which cause it to be difficult to identify the dynamic characteristic confidents of sliding bearing in time domain conditions.

Considering the periodicity or near time-invariance of these influencing factors, they can be eliminated by solving the equation in frequency domain condition after the Fourier transform.

(2) $$\left\{ \begin{array}{l} (k+j\omega c)\left[\begin{array}{l} {\bar{X}}_{2}(\omega )\\ {\bar{Y}}_{2}(\omega ) \end{array}\right]=\left[\begin{array}{l} \sqrt{2}/2\\ \sqrt{2}/2 \end{array}\right]{\bar{F}}_{1}(\omega )+\left[\begin{array}{l} -\sqrt{2}/2\\ \sqrt{2}/2 \end{array}\right]{\bar{F}}_{2}(\omega )+(m\omega^{2}-k_{0}-j\omega c_{0})\left[\begin{array}{l} \bar{X}(\omega )\\ \bar{Y}(\omega ) \end{array}\right]\\ (-m_{1}\omega^{2}+k_{1}+j\omega c_{1})\left[\begin{array}{l} {\bar{X}}_{1}(\omega )\\ {\bar{Y}}_{1}(\omega ) \end{array}\right]=(k+j\omega c)\left[\begin{array}{l} {\bar{X}}_{2}(\omega )\\ {\bar{Y}}_{2}(\omega ) \end{array}\right] \end{array}\right.$$

The common methods for applying exciting forces include the single frequency excitation method and multi-excitation method. In the multi-excitation method, two sinusoidal exciting forces with different frequencies are simultaneously applied on the test bearing in two vertical directions. Then the responses are plugged into the equation, and the Equation (2) is transformed as follows:

(3) $$\left\{ \begin{array}{l} (k-j\omega c)\left[\begin{array}{l} {\bar{X}}_{2}(\omega_{1})/{\bar{F}}_{1}(\omega_{1})\\ {\bar{Y}}_{2}(\omega_{1})/{\bar{F}}_{1}(\omega_{1}) \end{array}\right]=\left[\begin{array}{l} \sqrt{2}/2\\ \sqrt{2}/2 \end{array}\right]+(m{\omega_{1}}^{2}-k_{0}-j\omega_{1}c_{0})\left[\begin{array}{l} \bar{X}(\omega_{1})/{\bar{F}}_{1}(\omega_{1})\\ \bar{Y}(\omega_{1})/{\bar{F}}_{1}(\omega_{1}) \end{array}\right]\\ (k-j\omega c)\left[\begin{array}{l} {\bar{X}}_{2}(\omega_{2})/{\bar{F}}_{2}(\omega_{2})\\ {\bar{Y}}_{2}(\omega_{2})/{\bar{F}}_{2}(\omega_{2}) \end{array}\right]=\left[\begin{array}{l} -\sqrt{2}/2\\ \sqrt{2}/2 \end{array}\right]+(m{\omega_{2}}^{2}-k_{0}-j\omega_{2}c_{0})\left[\begin{array}{l} \bar{X}(\omega_{2})/{\bar{F}}_{2}(\omega_{2})\\ \bar{Y}(\omega_{2})/{\bar{F}}_{2}(\omega_{2}) \end{array}\right] \end{array}\right.$$

The Equation (3) is the measurement equation of the multi-excitation method. The masses, coupling stiffness and damping confidents are all known. The equation can be solved by plugging the amplitude ratio and the phase difference between the forces and displacements into it.

## 3. Constitution and Dynamic Calibration Method of the Measurement System

### 3.1. Constitution of the Measurement System

**Figure 3:**
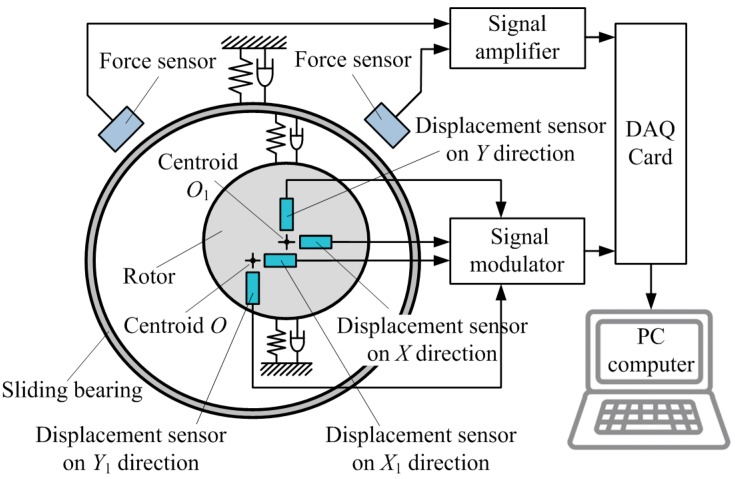
Constitution of the measurement system for sliding bearing.

According to Equation (3), the measurement system for dynamic characteristic coefficients of sliding bearing in the inversion test bed should contain four displacement test channels and two force test channels at least, which is shown in Figure 3.

The voltage signals of force sensors and displacement sensors are acquired by DAQ Card after being amplified or modulated, and are sent to the computer. Then the voltage signals will be transformed into force signals and displacement signals by test software.

### 3.2. Error Propagation Analysis of Test Signals

From the Equation (3) and Figure 3, the two physical quantities of force and displacement should be tested at first, and the force and displacement signals acquired are analyzed in frequency domain conditions. Then the stiffness and damping coefficients of the bearing can be identified by plugging the amplitude ratio and the phase difference between the forces signals and displacements signals into Equation (3). So the ratio between the displacement signal and force signal is defined as the transfer function of the sliding bearing.

(4) $$H\left(\omega \right)=\frac{X\left(j\omega \right)}{F\left(j\omega \right)}$$

where *H*(*ω*) is the transfer function of sliding bearing. It represents the relationships between the exciting force and displacement response of the bearing. The transfer function of sliding bearing is a plural flexibility, and its value is related to the frequency and amplitude of the exciting force. *F*(*jω*) is the real force signal, *X*(*jω*) is the real displacement response signal of the sliding bearing.

**Figure 4:**
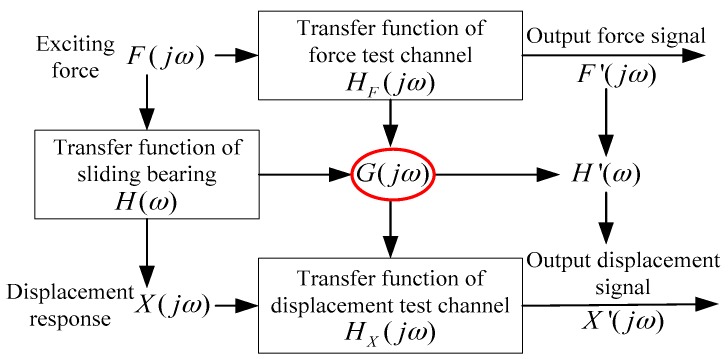
Error propagation and conversion of test signals in measurement system.

In the practical test process, the forces acting on the bearing by the exciter are transformed into voltage signals by force sensors. These signals are usually slight, and are amplified by the signal amplifier before being acquired by the DAQ Card. It is assumed that the transformation coefficient between the test force signal $F^{\prime }\left(j\omega \right)$ and the real force signal F(jω) is $H_{F}\left(j\omega \right)$, which is defined as the transfer function of the force test channel. It is shown in Figure 4.

The transfer function of the force test channel is related to the materials and structures of the sensor itself, and is the intrinsic property of the sensor. So the test signals can be converted to the real signals though the following equation:

(5) $$F\prime (j\omega )=F(j\omega )H_{F}(j\omega )$$

In a similar way, the relationship between the real displacement signal X(jω) and the test signal X′(jω) of the sliding bearing is as follows:

(6) $$X\prime (j\omega )=X(j\omega )H_{X}(j\omega )$$

where $H_{X}\left(j\omega \right)$ is the transfer function of displacement test channel.

Then the transfer function of the sliding bearing is deduced by jointing the Equations (4)–(6):

(7) $$H\left(\omega \right)=\frac{X\prime (j\omega )}{F\prime (j\omega )}\cdot \frac{H_{F}(j\omega )}{H_{X}(j\omega )}$$

It is defined as follows:

(8) $$\left\{ \begin{array}{l} G\left(j\omega \right)=\frac{H_{X}(j\omega )}{H_{F}(j\omega )}\\ H\prime \left(\omega \right)=\frac{X\prime (j\omega )}{F\prime (j\omega )} \end{array}\right.$$

and the transfer function of sliding bearing is converted to:

(9) $$H\left(\omega \right)=\frac{H\prime \left(\omega \right)}{G\left(j\omega \right)}$$

where G(jω) is the relative transfer function between the force test channel and displacement test channel. H′(ω) is the ratio between the test displacement signal and test force signal.

From Equation (9), if the G(jω) is known, then the transfer function of sliding bearing H(ω) can be acquired by the test signals F′(jω) and X′(jω). Theoretically, the transfer functions $HY_{F}\left(j\omega \right)$ and $H_{X}\left(j\omega \right)$ of force sensors and displacement sensors in the measurement system are all known. However, in fact, there are many other errors caused by elements and connection of components in the test channels except for sensors. The transfer function of each test channel is no longer known, and cannot even be detected individually. The purpose of the dynamic calibration for the measurement system in this paper is to detect the relative transfer function G(jω) by uniting the force test channel and displacement test channel synthetically. After the G(jω) is confirmed, the transfer function of sliding bearing H(ω) can be obtained by test signals F′(jω) and X′(jω) and Equation (9), and then the dynamic characteristic coefficients of sliding bearing can be identified by plugging H(ω) into Equation (3).

### 3.3. Dynamic Calibration Method of Measurement System

According the analysis above, the dynamic calibration of the measurement system is to acquire the transfer function G(jω) between the force test channel and displacement test channel, which usually proceeds by creating a calibration device with a known transfer function. It is assumed that the transfer function of the calibration device is $H_{0}\left(j\omega \right)$, on which the exciting force F(jω) is acted. The displacement is X(jω). The force signal and displacement signal obtained by the measurement system are F′(jω) and X(jω). Then the transfer function between the test force and displacement is as follows:

(10) $$H_{0}\prime (j\omega )=\frac{X\prime (j\omega )}{F\prime (j\omega )}=\frac{X(j\omega )H_{X}(j\omega )}{F(j\omega )H_{F}(j\omega )}=\frac{X(j\omega )}{F(j\omega )}\cdot \frac{H_{X}(j\omega )}{H_{F}(j\omega )}=H_{0}(j\omega )\cdot G(j\omega )$$

where $H_{0}\left(j\omega \right)$ is the transfer function of calibration device. It is a known physical quantity. $H_{0}\prime \left(j\omega \right)$ can be obtained by the test force and displacement signals.

According to Equation (7), the transfer function of measurement system is as follows:

(11) $$G(j\omega )=\frac{H_{0}\prime (j\omega )}{H_{0}(j\omega )}$$

As the dynamic characteristic confidents test is carried out under frequency domain conditions, the transfer function and test signals are usually expressed by amplitude and phase. It is assumed that the test force and displacement signals obtained in calibration are as follows:

$$\left\{ \begin{array}{l} F\prime (j\omega )=A_{F}(\omega )e^{j(\omega t+\varphi_{F}(\omega ))}\\ X\prime (j\omega )=A_{X}(\omega )e^{j(\omega t+\varphi_{x}(\omega ))} \end{array}\right.$$

If the transfer function of measurement system is: $G(j\omega )=A(\omega )e^{j\left(\varphi (\omega )\right)}$, and the transfer function of calibration device is: $H_{0}(j\omega )=A_{H0}(\omega )e^{j\left(\varphi_{0}(\omega )\right)}$.

According to the Equations (10) and (11), the transfer function of measurement system can be expressed as:

(12) $$G(j\omega )=\frac{H_{0}\prime (j\omega )}{H_{0}(j\omega )}=\frac{X\prime (j\omega )}{F\prime (j\omega )}\cdot \frac{1}{H_{0}(j\omega )}=\frac{A_{X}(\omega )}{A_{H0}(\omega )A_{F}(\omega )}e^{j(\varphi_{x}(\omega )-\varphi_{F}(\omega )-\varphi_{0}(\omega ))}$$

So the amplitude and phase of transfer function of measurement system are as follows:

(13) $$\left\{ \begin{array}{l} A(\omega )=\frac{A_{X}(\omega )}{A_{H0}(\omega )A_{F}(\omega )}\\ \varphi (\omega )=\varphi_{X}(\omega )-\varphi_{F}(\omega )-\varphi_{0}(\omega ) \end{array}\right.$$

## 4. Dynamic Calibration Device and Experiment

### 4.1. Dynamic Calibration Device

**Figure 5:**
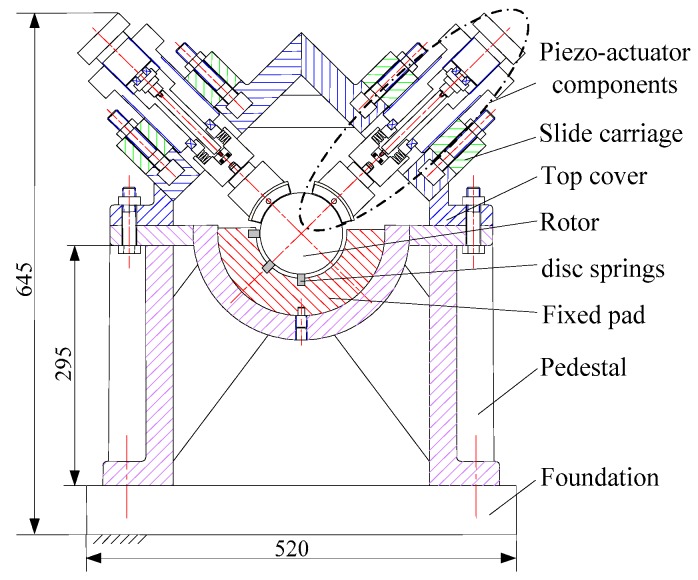
Scheme of dynamic calibration device.

The scheme of dynamic calibration device proposed in this paper is shown in Figure 5. The embedded loading technique is introduced into this scheme to act the static and dynamic forces on the rotor. The embedded loading technique is implemented by piezo-actuator, which can generate dynamic loads, such as harmonic, square wave and pulse forces.

**Figure 6:**
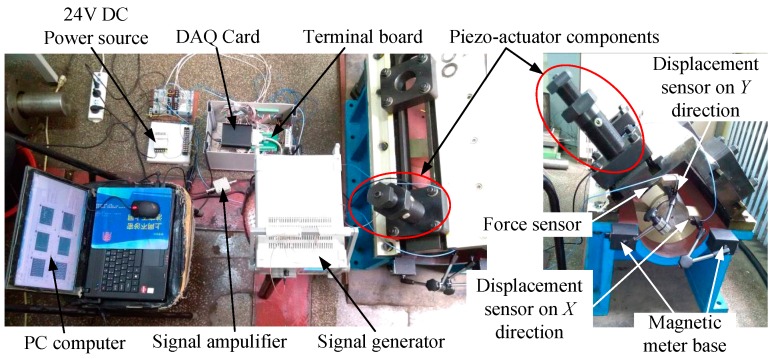
Calibration device and the test points placement.

A set of loading devices of piezo-actuator is installed on the −45° direction of the test bed. The fixed pad is a slot with two grooves on the ±45° direction to set the disc spring components, of which the stiffness is 10^6^ ~ 10^7^ N/m order of magnitude. The main parameters of the disc spring are as follows: the inner diameter is 25.4 mm, the outer diameter is 50 mm and the thickness is 3 mm. The stiffness of disc spring components is 6.2 × 10^7^ N/m. Two eddy current sensors are respectively set in horizontal and vertical directions. The loading forces are measured by the force sensor set between the tilting pad and the bearing base. The calibration device and the test points placement are shown in Figure 6.

**Figure 7:**
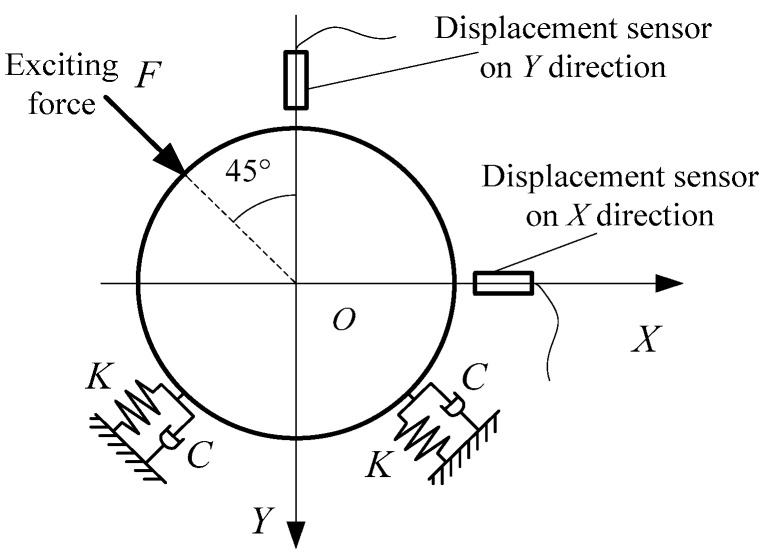
Dynamical model of calibration device.

The dynamical model of calibration device is shown in Figure 7. The sinusoidal excitation generated by piezo-actuator acts on the rotor through a loading pad. Because of the stiffness of the disc springs and the damping of the junction surfaces, the differential equation of rotor’s motion is as follows:

(14) M[X¨Y¨]+C[X˙Y˙]+K[XY]=[22−22]F

where *M* is the mass of rotor; *K* is the stiffness of disc springs on the direction of the exciting force; *C* is the damping on the direction of the exciting force.

After Fourier transform:

(15) (K−Mω2+jωC)[X¯(jω)Y¯(jω)]=[22−22]F¯(jω)

So the transform functions between the exciting force and two displacements are as follows:

(16) $$\left\{ \begin{array}{l} H_{FX}\left(j\omega \right)=\frac{\bar{X}\left(j\omega \right)}{\bar{F}\left(j\omega \right)}=\frac{1}{\sqrt{2}\left(K-M\omega^{2}+j\omega C\right)}\\ H_{FY}\left(j\omega \right)=\frac{\bar{Y}\left(j\omega \right)}{\bar{F}\left(j\omega \right)}=-\frac{1}{\sqrt{2}\left(K-M\omega^{2}+j\omega C\right)} \end{array}\right.$$

Their amplitudes and phases are as follows:

(17) $$\begin{array}{ccc} \left\{ \begin{array}{l} A_{HX}\left(\omega \right)=\frac{1}{\sqrt{2\left[{\left(K-M\omega^{2}\right)}^{2}+{\left(\omega C\right)}^{2}\right]}}\\ \varphi_{HX}\left(\omega \right)=\arctan\left(\frac{\omega C}{M\omega^{2}-K}\right) \end{array}\right. & ; & \left\{ \begin{array}{l} A_{HY}\left(\omega \right)=\frac{1}{\sqrt{2\left[{\left(K-M\omega^{2}\right)}^{2}+{\left(\omega C\right)}^{2}\right]}}\\ \varphi_{HY}\left(\omega \right)=180{}^{\circ}+\arctan\left(\frac{\omega C}{M\omega^{2}-K}\right) \end{array}\right. \end{array}$$

Plugging Equation (17) into Equation (13), the amplitudes and phases of the transform functions between the force test channel and two displacement test channels of the measurement system for dynamic characteristic coefficients are as follows:

(18) $$\left\{ \begin{array}{l} {}[\begin{array}{l} A_{GX}\left(\omega \right)=\sqrt{2\left[{\left(K-M\omega^{2}\right)}^{2}+{\left(\omega C\right)}^{2}\right]}\cdot \frac{A_{X}(\omega )}{A_{F}(\omega )}\\ \varphi_{GX}\left(\omega \right)=\varphi_{X}(\omega )-\varphi_{F}(\omega )-\arctan\left(\frac{\omega C}{M\omega^{2}-K}\right) \end{array}\\ {}[\begin{array}{l} A_{GY}\left(\omega \right)=\sqrt{2\left[{\left(K-M\omega^{2}\right)}^{2}+{\left(\omega C\right)}^{2}\right]}\cdot \frac{A_{Y}(\omega )}{A_{F}(\omega )}\\ \varphi_{GY}\left(\omega \right)=\varphi_{Y}(\omega )-\varphi_{F}(\omega )-180{}^{\circ}-\arctan\left(\frac{\omega C}{M\omega^{2}-K}\right) \end{array} \end{array}\right.$$

### 4.2. Analysis of Experiment Results Analysis

#### 4.2.1. Damping Identification of Calibration Device

**Figure 8:**
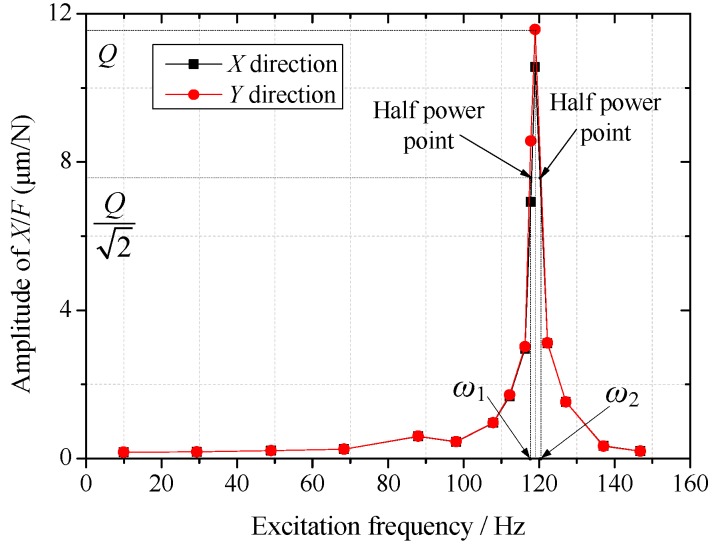
Amplitude-frequency curves of the rotor vibrations on the X and Y directions.

There is low damping in the junction surfaces of disc springs. In order to eliminate the influence of the damping on the calibration data, the frequency scanning test is implemented on the calibration device. The exciting forces of frequency 10 Hz–150 Hz act on the rotor by piezo-actuator. The amplitude-frequency curves of the rotor vibrations on the X and Y directions acquired though the measurement system are shown in the Figure 8.

Because the calibration device is a system of high stiffness and low damping, there exists a significant resonance phenomenon. The system damping can be identified though Equation (19):

(19) $$C=M\left(\omega_{2}-\omega_{1}\right)$$

where *M* is the mass of rotor, and *M* = 7.32 kg; *ω*_1_ and *ω*_2_ are the two corresponding frequencies of the half-power points on the curve. The system damping identified on the two directions are equal, and *C* = 146 N/(m∙s^–1^).

#### 4.2.2. Calibration Data of the Measurement System under Different Frequencies

**Figure 9:**
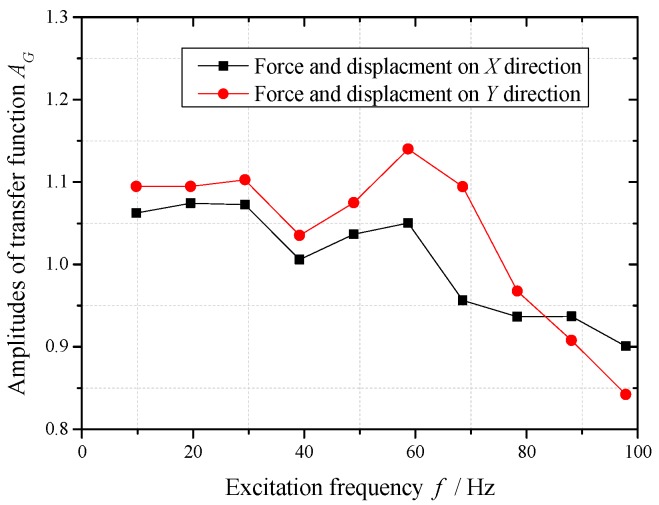
Amplitudes of transfer function of measurement system under different frequencies.

**Figure 10:**
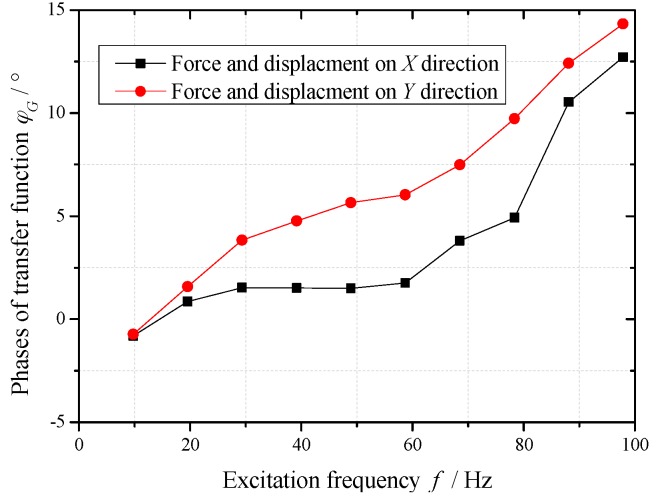
Phases of transfer function of measurement system under different frequencies.

The exciting forces of 10 Hz–100 Hz frequencies act on the calibration device, which is repeated five times. The values of mass, stiffness and damping are plugged into Equation (18) along with the test forces and displacements. Taking an average of the five tests, the calibration data of measurement system under different frequencies are shown in Figure 9 and Figure 10.

In the excitation frequency range of 10 Hz–100 Hz, the amplitudes of the transfer function are in the range of 1% ± 15%, and the phases increase along with the increasing of excitation frequency. The amplitudes and phase differences of the transfer function between force test channel and two displacement test channels are small (amplitudes difference 5%, phases difference 5°).

In the frequency range of 10 Hz–30 Hz, the amplitude and phase of test results using this measurement system need to be amended respectively less than 10% and 3°. The calibration data in this frequency range can support the dynamic characteristics test of sliding bearing in this frequency range well.

It is important to note that the excitation frequencies should be equal to the corresponding frequencies of the data points in Figure 9 and Figure 10, when this measurement system is used to identify the dynamic characteristic coefficients of a sliding bearing. Moreover, the test results are amended by the corresponding amplitudes and phases in calibration data. Otherwise, other errors will be introduced by interpolation of calibration data, and the reliability of analysis results will decline.

#### 4.2.3. Identification Experiment of Dynamic Characteristic Coefficients Using the Calibration Data

In order to verify the calibration data of the measurement system, the simulated experiment is implemented on the calibration device. The dynamic characteristic coefficients of the sliding bearing are simulated by the stiffness of disc springs and the damping of junction surfaces, and the exciting forces act on the rotor by piezo-actuator, which forms a simulated erected test bed for the dynamic characteristic coefficients of the sliding bearing [36]. The dynamic model is shown in Figure 7, and the differential equations of motion are as follows:

(20) m[X..Y..]+c[X.Y.]+k[X˙Y]=[2222]F1+[−2222]F2

When using the multi-excitation method, the measurement equations are as follows:

(21) {(k−mω12+jω1c)[X(ω1)/F1(ω1)Y(ω1)/F1(ω1)]=[2222](k−mω22+jω2c)[X(ω2)/F2(ω2)Y(ω2)/F2(ω2)]=[−2222]

Acting the exciting forces of frequency 20 Hz and 30 Hz on the test bed respectively, the exciting forces and the displacements acquired by the measurement system are shown in Table 1.

Without considering the test errors of the measurement system, the test data of forces and displacements are plugged into the measurement Equation (18) of the erected test bed. The stiffness and damping coefficients of the simulated bearing are identified. After the measurement system is calibrated, the test errors of the measurement system are separated from the test data using the calibration data in Figure 9 and Figure 10. Then the stiffness and damping coefficients of the simulated bearing are identified though the amended data. The comparisons between identification results and the given values are shown in Table 2.

According to the comparisons in Table 2, the identification results of dynamic characteristic coefficients are obviously different form the given values when the measurement system is not calibrated. The identification errors of principal stiffness are respectively −8.95% and −12.02%. The identified values of principal damping are negative, and the absolute value is 3–7 times the given values. These errors are too large for the research on dynamic behavior of sliding bearing, because they may cause a very big deviation of the research result.

However, the identification results of principal stiffness using the calibration data of the measurement system are close to the given values, of which the identification errors are respectively −2.36% and −3.58%. The identification errors of principal damping are slightly larger than the given values, and the errors are 18.58% and 7.48%. These identification errors are in the permitted ranges. So it is verified that the calibration data of the measurement system in the frequency range of 10 Hz–30 Hz can support the dynamic characteristics test of sliding bearing well.

## 5. Conclusions

(1) For the measurement system for dynamic characteristics coefficients of sliding bearing, a novel dynamic calibration method by jointly calibrating multiple test channels is proposed in this paper. The calibration device contains a spring-mass system, which can simulate the dynamical characteristics of the sliding bearing.
(2) The dynamic calibration device, including the piezo-actuator, force sensor and eddy current displacement sensor is designed and built. The dynamic calibration experiment in a wide frequency range simulating the bearing stiffness by disc springs is implemented. The experimental results show that the amplitude errors of this measurement system are small (less than ±15%) in the frequency range of 10 Hz–100 Hz, and the phase errors increase along with the increasing of frequency.
(3) The simulated experiment of dynamic characteristics coefficients identification is implemented on this calibration device using the calibration data of this measurement system in the frequency range of 10 Hz–30 Hz. The identification errors of principal stiffnesses are respectively −2.36% and −3.58%, and the identification errors of principal dampings are 18.58% and 7.48%, which are all far smaller than the identification errors without calibration. It is preliminarily verified that the calibration data in this frequency range can support the dynamic characteristics test of sliding bearing in this frequency range well.

## Figures and Tables

**Table 1:** Exciting forces and the displacements test by multi-excitation method.

Physical Quantities	First Excitation			Second Excitation		
	Exciting Force *F*_1_	Displacement on *X* Direction	Displacement on *Y* Direction	Exciting Force *F*_2_	Displacement on *X* Direction	Displacement on *Y* Direction
Units	N	μm	μm	N	μm	μm
Frequencies	19.54	19.6	19.53	29.33	29.32	29.32
Amplitudes	22.1	4.54	4.72	90.87	16.95	17.52
Phases	−83.82	−83.43	−82.72	150.9	−27.85	154.56

**Table 2:** Comparisons between identification results of dynamic characteristic coefficients and the given values.

Simulated Dynamic Characteristic Coefficients	Units	Given Values	Identification Results			
			Not Calibrated	Error %	After Calibrated	Error %
*k_xx_*	(×10^6^) N/m	4.09	3.724	−8.95	3.993	−2.36
*k_xy_*	(×10^6^) N/m	0	−0.055	-	−0.071	-
*k_yx_*	(×10^6^) N/m	0	−0.075	-	−0.103	-
*k_yy_*	(×10^6^) N/m	4.09	3.598	−12.02	3.944	−3.58
*c_xx_*	N/(m∙s^−1^)	146	−347.463	−337.99	173.126	18.58
*c_xy_*	N/(m∙s^−1^)	0	167.316	-	68.165	-
*c_yx_*	N/(m∙s^−1^)	0	343.945	-	87.227	-
*c_yy_*	N/(m∙s^−1^)	146	−866.497	−693.49	156.923	7.48
